# Task design for crowdsourced glioma cell annotation in microscopy images

**DOI:** 10.1038/s41598-024-51995-8

**Published:** 2024-01-23

**Authors:** Svea Schwarze, Nadine S. Schaadt, Viktor M. G. Sobotta, Nicolai Spicher, Thomas Skripuletz, Majid Esmaeilzadeh, Joachim K. Krauss, Christian Hartmann, Thomas M. Deserno, Friedrich Feuerhake

**Affiliations:** 1 Department of Neuropathology, Institute for Pathology, Hannover Medical School, Hannover, Germany; 2 Peter L. Reichertz Institute for Medical Informatics of TU Braunschweig and Hannover Medical School, Braunschweig, Germany; 3 Department of Neurology, Hannover Medical School, Hannover, Germany; 4 Department of Neurosurgery, Hannover Medical School, Hannover, Germany; 5 Institute of Neuropathology, University Clinic Freiburg, Freiburg, Germany

**Keywords:** CNS cancer, Translational research, Cancer microenvironment, Computational science, Image processing

## Abstract

Crowdsourcing has been used in computational pathology to generate cell and cell nuclei annotations for machine learning. Herein, we broaden its scope to the previously unsolved challenging task of glioma cell detection. This requires multiplexed immunofluorescence microscopy due to diffuse invasiveness and exceptional similarity between glioma cells and reactive astrocytes. In four pilot experiments, we iteratively developed a task design enabling high-quality annotations by crowdworkers on Amazon Mechanical Turk. We applied majority or weighted vote and validated them against ground truth in the final setting. On the base of a YOLO convolutional neural network architecture, we used these consensus labels for training with different image representations regarding colors, intensities, and immmunohistochemical marker combinations. A crowd of 712 workers defined aggregated point annotations in 235 images with an average $F_1$ score of 0.627 for majority vote. The networks resulted in acceptable $F_1$ scores up to 0.69 for YOLOv8 on average and indicated first evidence for transferability to images lacking tumor markers, especially in IDH-wildtype glioblastoma. Our work confirms feasibility of crowdsourcing to generate labels suitable for training of machine learning tools in the challenging and clinically relevant use case of glioma microenvironment.

## Introduction

Crowdsourcing addresses the urgent need for training data in machine learning (ML), as it has been shown that crowdlabels can be feasible to train convolutional neural networks (CNNs)^1–3^. It considers the collaborative solution of problems by several workers with heterogeneous domain knowledge, in the format of participatory online activity^4^. In this constellation, neither the contributors’ educational backgrounds, nor their environmental conditions are known, as there is no direct contact to the crowd. Therefore, strategies for successful task design include a focus on simple tasks and qualification phases with performance tests^5^. Crowd instructions should use simple English without complex scientific terms and provide illustrated or animated explanations in the qualification phase. Further, tasks should allow timely completion, preferably within less than 10 min^6^. It is recommendable to test task design in pilot studies to prove its reliability. In the case of paid contributions, compensation schemes can influence performance^7^ and thus may have impact on quality of results.

Crowdsourcing has been widely used in cancer research^8–10^. In microscopic images, crowds were asked for scoring of unambiguously stained cells or annotation of cell nuclei^11–13^, representing tasks of low complexity. Expanding crowdsourcing towards more complex problems requires several considerations. First, semantic content and visual appearance have a strong influence on how crowdworkers perceive task complexity^14^. Secondly, complexity strongly increases with higher number of classes; e.g., school pupils annotated cells in a competitive format with levels from “mild” (annotating a single cell type) step-wise adding further cell types up to “supercharger” level, with decreasing accuracy in higher levels^15^. A third type of task complexity concerns required skills for image annotation, such as delineation of anatomical or pathologically altered structures. This typically requires specific strategies to achieve satisfying results including “educated” crowds with prior knowledge (e.g., medical students) and/or an advanced work setting, such as direct contact between requester and crowdworker^16–18^.

Accurate cell detection is critical to analyze brain tumor microenvironments^19^. However, it is difficult to distinguish reactive astrocytes (cells from central nervous system) from almost identically-looking tumor (glioma) cells in this use case. Tumor cells can resemble their normal counterparts (astrocyte-like differentiation) and non-malignant astrocytes may assume phenotypes resembling malignant cells (reactive astrocytosis)^20^. This is in contrast to, for example, solid epithelial tumors, where de-differentiated malignant cells may share some morphological features with the tissue of origin (e.g., gland-like structures), but pre-existing surrounding tissues do not significantly change their characteristics towards a malignant appearance. In the CNS, reactive astrocytosis can acquire morphological features resembling malignant giant cells, even in inflammatory conditions like multiple sclerosis^21^. Given this exceptional overlap between pre-existing reactive and truly malignant components of glioma microenvironment, the combination of immunohistochemical markers and the size and quality of training data for ML are especially important in this use case. In the context of crowdsourcing, this shifts cell labeling to a complex challenge, with the need to integrate information from multiple color channels with morphological features. The required markers can be stained and visualized in so-called multiplex immunofluorescence images (MSIs)^22^. MSI s in cancer research provide the advantage of combining in-depth information about cellular subtypes by several markers with spatial information on cell level. This allows distance metrics and neighborhood analyses to study possible cell-cell-interactions^23^. In these fluorescence images 4^′^,6-Diamidin-2-phenylindol (DAPI) is widely used to stain nuclei of all cells and therefore used in segmentation algorithms. Deep learning is frequently used for cell detection in MSIs, e.g., to study co-registered slides^24^ or spatial relations^25^. Greenwald et al. introduced a cell phenotyping iteratively trained on crowdsourced and expert-corrected data for several cell types in multiple tumor and tissue types^26^. However, this study lacks brain tumors and therefore focuses on cell types that could be easily recognized based on staining. In brain context, $\upalpha$-thalassemia/mental retardation syndrome X-linked protein (ATRX) is a nuclear marker in non-neoplastic cells, but lost in the tumor cells of many Isocitrate dehydrogenase 1 (IDH1)-mutated astrocytomas and glial fibrillary acidic protein (GFAP) is expressed by cells with astrocytic origin^27^. Using these markers, tumor cells can be distinguished from astrocytes. Whereas tumor cell nuclei are negative for ATRX and possibly surrounded by GFAP, the nuclei of astrocytes are positive for ATRX and also surrounded by GFAP. Thereby, astrocytes show mostly a star shaped character. The morphology is also important to distinguish these two cell types from others like neurons with larger ATRX-positive nuclei.

In this work, we developed a task design for advanced cell annotations in MSIs of IDH1-mutated high-grade astrocytoma for paid crowds. Addressing the above mentioned complexity and quality assurance, we analyzed the image size, crowd size, and task description in pilot experiments. In order to evaluate the reliability of crowd annotations based on the developed task design, we used them for training YOLO-based CNNs and tested them against truth provided by experts. We further considered the transfer to IDH-wildtype glioblastoma without ATRX-loss. Our major contributions are

- distinguishing glioma cells from similar astrocytes based on both morphology and staining,
- developing an iteratively optimized task design for acquisition of feasible annotations,
- training a CNN transferable to different glioma types, and thereby
- overcoming annotation bottleneck in glioma microenvironment studies.

## Related work

Since the first formal description of crowdsourcing in 1907^28^, traditional crowdsourcing methods are applied to solve specific tasks with the main purpose to directly use the crowdlabels. Therefore, quality control measures like confidence scores or scattered gold standard are required to eliminate noisy labels^16^. As pathology is a domain characterized by highly specialized medical expertise, most publications focus on showing reliability^8^. A representative example for crowdsourcing applications showed feasibility by comparing the crowd against experts for a small subset in the context of estrogen receptor scoring in breast cancer, requiring thresholding and distinction of relevant cancer cells from normal gland cells, immune cells, or artifacts^29^. Individual examples like these, in line with a growing body of evidence beyond pathology, as recently reviewed by Zhang et al., support a major role of platforms, adapted task design, instructions, fairness of compensation/rewards, and motivational aspects on the solver/participant side as emerging research topics for crowdsourcing in knowledge-intensive tasks^30^.

Recent developments to further increase scalability aim at integrating crowdlabels into machine learning frameworks to improve performance of networks. Examples include segmentation of cell nuclei with a VGG16 trained on crowdlabels^3^, combination of a network trained on crowdlabels with an network based on experts^2^, or labeling of mitoses predicted by a CNN trained on expert annotations, with subsequent use of expectation-maximization-based aggregated crowdlabels to fine-tune the network^1^. Alternatively, Greenwald et al. propose an iterative approach between training, crowdsourced correction, expert correction, and re-training^26^. In contrast, answer aggregation is commonly used to reduce the influence of noisy labels. For example, label augmented and weighted majority voting (LAWMV), in which each label is weighted based on the label of close data set instances, is shown to outperform other methods like majority vote^31^. Several approaches are based on expectation-maximization^32^, e.g., an additional layer, called “crowd layer”, was included by Rodrigues et al. to directly utilize all individual crowdlabels using backpropagation^33^. Wei et al. propose a novel U-Net, which connect all labels from all annotators and handle them as union^34^. Detailed comparisons between label separation and different aggregation methods showed that among other things task design and crowd size have a strong impact on the performance of method^35^.

Related work in the context of multiplexed stainings is largely focused on staining information only, not primarily considering morphological or contextual aspects. Several work focus on marker intensity thresholds, e.g., manually selected by experts and then used to train a classifier^19^. Other methods include detection of lymphocytes or macrophages using U-Net and YOLOv2 as transfer learning approach from brightfield images^25^. Bounding box detection of astrocytes and other normal brain cell populations could be solved by a Faster RCNN and phenotyping them based on a Capsule Network^24^. However, the appearance of cells in malignant altered tissue is completely changed, such that phenotyping purely based on staining information is often not satisfying, especially to distinguish between reactive astrocytes and tumor cells.

## Methods

We performed four pilot experiments to iteratively adopt the task design. Based on the final task design, further experiments provided crowdsourced labels for training. We considered different color schemes for the CNNs.

### Crowdsourcing

#### Setting

Crowdworkers were asked to click on certain cells in MSIs on Amazon Mechanical Turk (AMT)^36^. Therefore, we extensively revised a website, which was developed for crowdsourced delineation in human eyes^37^. On this website, the basic concept (task description, qualification phase with performance test) we needed was already implemented: Crowdworker can navigate with next respectively previous button or go directly back to the task description at any time. Workers were asked to set point annotations by left-clicking for only one class. These points could be removed with right-clicking or short key. At least one point had to be set per image, otherwise the image was displayed again later on. Furthermore, workers could check their annotations in a mask with a transparent image overlay. The order of the images of one task could be different between crowdworkers, since the image that was edited least often was displayed first. Only the last image was the same for all workers as it was used for quality control (see “Answer aggregation” section). If they were not finished after 45 minutes, the task was terminated automatically. Obviously, the number of labels needed to train a CNN is too large for a single worker. At the same time, a minimum number of workers per MSI is necessary for satisfying quality. Therefore, we divided the entire AMT crowd of 712 workers into subgroups. Each subgroup annotated other MSIs. We uploaded 235 MSIs (74 for tumor tasks and 163 for astrocyte tasks) to AMT. Due to task complexity and correspondingly required enlarged tutorial and qualification phase, a task was designed for 15–20 min and paid with $2. To reduce the risk of technical errors, we provided them at different days. Following the recommendation to divide the task into smaller ones^38^, we split the job in two independent tasks for astrocytes and tumor cells. Due to the common imbalance that many more tumor cells than astrocytes occur, the crowd should annotate more MSIs for the astrocyte task. The proportion of both cell types differs depending on the region within the glioma. Thus, we used different MSIs for both tasks. For crowdsourcing, the MSIs were cut into tiles with an overlap of 30 pixels to ensure that cells at the boarder were entirely displayed. In postprocessing, the crowdlabels were stitched together to the size of the MSI.

#### Task design

The instructions contained plain text with task description and its medical relevance intended to increase workers’ motivation. The different cell types were explained by example images (14 for astrocytes and 9 for tumors showing some representative cells each) together with the same image in which the target cells were delineated. Subsequently, an obligatory qualification phase followed which comprised three MSI-tiles, which had to be annotated with a positive predictive value (PPV, precision) of at least 0.8 before being able to proceed to the main task. To increase clarity, we reduced the markers to the most essential ones (referred to as Var1): GFAP in blue, ATRX in red, and DAPI in white; i.e., the crowd was asked to detect astrocytes based on red nuclei with a mostly star-shaped blue surrounding and tumor cells based on white nuclei. We chose this scheme by considering common color weaknesses. In clinical routine, a marker for IDH1 (R132H) is commonly used to visualize the tumor cells. We also stained this marker, but decided to not include its channel for crowdsourcing due to the following reasons. An additional color would further increase the complexity. Especially, the overlap with GFAP requires tools to switch markers on and off, in which an annotation becomes difficult for non-experts. Therefore, we focus on ATRX as a nuclear marker.

#### Pilot experiments

To reveal the optimal tile size (PE1), volunteers consecutively annotated the same MSI twice, once with small tiles (MSI cut into 48 tiles) and once with large tiles (cut into four tiles). Since the astrocyte density can be low, tiles without any astrocyte can occur e.g., in the tumor core. Low densities could lead to false positives as workers need to label at least one cell for technical reasons and probably expect several target cells in an image. Therefore, we selected for PE1 MSIs with at least 22 astrocytes. To control the possible influence on the second annotations by remembering the first annotation, we asked the workers in a third step immediately after both annotations to label a couple of images. Shown were a mixture of 10 images, five from the annotation set and five new ones. The workers should select whether they know this image or not. An expert did the same, but additionally re-labeled all cells with discrepancies. Further focus was on (i) a suitable crowd size (PE2), (ii) the time required to perform a task (PE3), and (iii) presentation of the task to enable fast understanding (PE4). We evaluated feedback based on questionnaires or direct contact in PE1–3 or analyzed the received crowdlabels in PE1, PE2, and PE4.

**Table 1:** We iteratively established the task design to ensure the quality of crowd annotations.

Pilot experiment	Crowd	Target parameter	Number of MSIs
PE1	18 volunteers	Tile size	13
PE2	25 high school students	Crowd size, instruction	4
PE3	10 volunteers	Number of MSIs, instruction	4
PE4	35 Amazon Mechanical Turk workers	Instruction	8

Therefore, we performed pilot experiments with volunteers, 10th grade students of a German cooperative comprehensive school, and a paid crowd (in this Table).

#### Answer aggregation

We aggregated all individual labels using majority vote (MV) and weighted vote (WV). Besides the tiles, all workers should edit a so-called ground truth-image (518 $\times$ 438 pixels for tumor task with 38 tumor cells, 878 $\times$ 940 pixels for astrocyte task with 7 astrocytes), based on which they were compared with an expert. This image was shown as last one of the task and the special meaning of it was unknown for the workers. For WV, a user’s weight was 1.00 if he correctly labeled all cells in the ground truth-image. A value of 0.20 was subtracted for each wrong cell with a minimum value of 0.05 for the final weight.

#### Feasibility

We measured Fleiss’ $\kappa$ to assess the inter-rater reliability between non-experts and experts. As a benchmark, three experts familiarized themselves with the staining on four MSIs, aligned each other in a consensus meeting, and afterwards independently annotated four new MSIs.

The crowd’s quality was evaluated on the above mentioned ground truth-image and further, so-called ground truth-tiles with expert labels (five tiles for tumor task of 518 $\times$ 438 pixels with 96 tumor cells, four tiles for astrocyte task of 940 $\times$ 878 pixels with 23 astrocytes) located in the center of MSIs scattered over the entire task. For this, true positives, false positives, and false negatives based on point-annotations were counted together in all ground truth-tiles to measure the true positive rate (TPR, sensitivity, recall), the PPV, and the $F_1$ score (Dice’s coefficient) as commonly defined.

### Machine learning

We used YOLO^39^, a widely applied architecture^40^ in version YOLOv5 and the currently latest version YOLOv8^41^. These models were pretrained on the Common Objects in Context data set^42^. For comparison, we applied a Faster RCNN architecture^43^. The bounding boxes for the training were automatically extracted from the point-based labels in a two-tiered approach. First, the image background was removed based on the lower intensity of the background-pixel as observed in the corresponding histogram. Secondly, the outlines of a cell were segmented by a flood-fill algorithm, expanding the labeled nuclei by either rejecting or including neighboring pixels based on contrast and color. Since astrocytes and tumor cells were labeled in different images, we generated training images that simultaneously marked both cell types in one image. Two preliminary models, each trained on one cell type, were utilized to generate the consolidated training data. We used 1000 epochs for training with at batch size of 7 performed on GPU Nvidia RTX 3080Ti in about 4 h per variant. We used a confidence threshold of 0.25 for testing and performed it on a CPU with 64 GB RAM. An estimation of the complexity of the single steps in the process is given in Supplementary Table 1.

**Figure 1:**
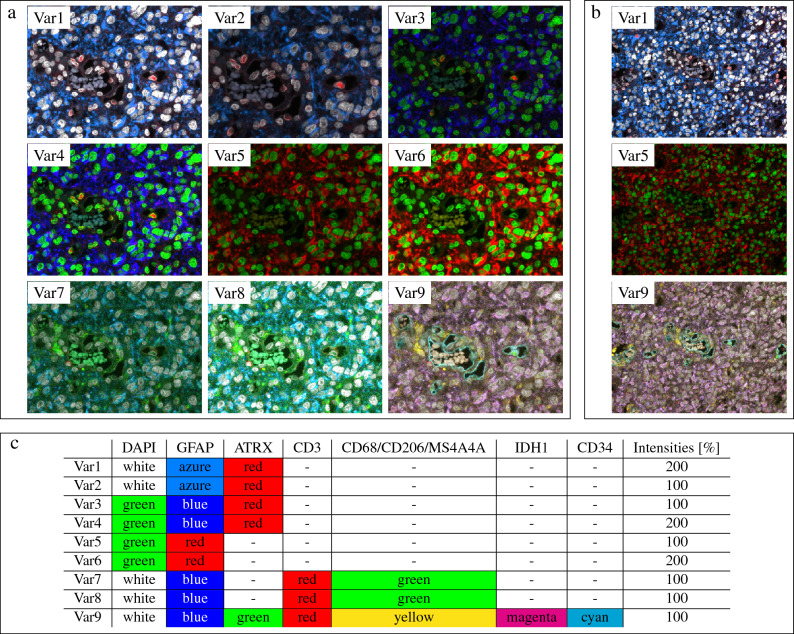
Image representation for machine learning variants. (**a**) Smaller tile. (**b**) Larger tile. Var1–4: markers DAPI, ATRX, and GFAP in different colors and intensities. Var5–6: DAPI and GFAP (but, without ATRX). Var7–9: not required markers added. (**c**) overview of the colors and intensities.

We considered nine variants for ML training (Fig. 1): Var1–Var4 included ATRX, GFAP, and DAPI in different colors or intensities. Var5–Var6 were reduced to GFAP and DAPI channels only. Lacking the information on ATRX expression, this resembles the situation in IDH1 wildtype glioblastoma, where ATRX expression cannot be part of cell classification. Var7–9 further added markers for cell types out of interest. Thereby, Var9 contained all stained markers. We trained and validated nine CNNs with consolidated data based on the aggregated crowdlabels (27 of the MSIs were used as validation set). The independent test set included expert annotations of 43 MSIs. It contained 11,571 annotated tumor cells and 322 astrocytes. As the number of cells in a single MSI could be small, we combined all cells over the entire test set to measure TPR, PPV, and $F_1$ score. For these measurements, we used an intersection over union of at least 0.35 based on the predicted and ground truth bounding boxes. In addition, we measured the average precision (AP50, AP@$[0.5:0.05:0.95]$).

### Material

The data set contained 59 brain tumor specimens from 43 patients. Thirty-three of these patients were considered for crowdsourcing and 19 patients in the ML test set. Paraffin sections were stained with the above mentioned markers (DAPI, ATRX, GFAP, IDH1) and additional markers for macrophages (MS4A4A, CD68, CD206), T-cells (CD3), and vessels (CD34). In the scanned slides, we randomly selected regions ranging form tumor core to adjacent normal tissue. These regions were multispectral color deconvoluted at a resolution of 0.25 µm/pixel (Vectra Polaris, Akoya Biosciences), resulting in MSIs of 1860 $\times$ 1396 pixels. To reduce bias, both data sets, the crowdsourcing and the ML test set cover different staining batches and different regions within the tissue. We used 298 MSIs in total. The use of anonymized tissue samples for digital pathology analyses was approved by the institutional review board of Hannover Medical School in accordance with the 1964 Helsinki declaration and its later amendments or comparable ethical standards (approval number 6960-2015 and 707-2013). The approved workflow includes obtaining informed consent from all subjects contributing anonymized tissue samples.

## Results

First, we present the results of the pilot experiments and explain how this led to the final task design. Then, we describe the measured reliability of crowds in further experiments and the validation of the nine CNNs trained on these crowdlabels.

### Crowdsourcing

#### Pilot experiments

In order to account for task complexity, we used an iterative design concept composed of four consecutive pilot experiments (PE1–PE4; Table 1). In experiment PE1, we investigated the tile size of the MSIs. PE1 showed no clear preference of the users based on a questionnaire addressing this aspect and resulted in a MV-$F_1$ score of 0.78 for smaller and of 0.70 for larger tiles, supporting an advantage of smaller tiles. Since cell density tends to be higher for tumor cells than for astrocytes, we used a larger tile size for astrocyte (MSIs cut into four tiles) and an intermediate size (MSIs cut into 16 tiles) for tumor tasks. This helped to reduce the risk of false positives due to low cell densities. We also tested the possibility of a bias from repeatedly annotating identical images in an experiment. Thereby, most of the workers were uncertain with their decision based on questionnaire. They correctly predicted whether they saw this image before for 2.1 out of five known and 3.4 out of five unknown images on average. That showed crowdworkers and experts did not recognize already annotated images.

**Figure 2:**
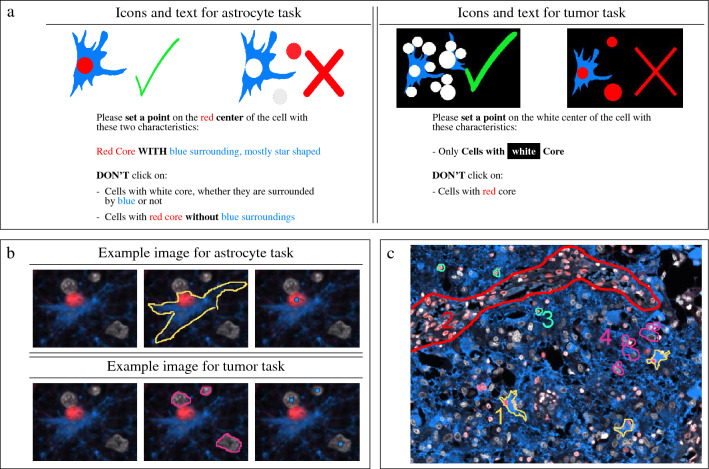
Crowd instruction. (**a**) Icons and text as information material. (**b**) Example image (first column for original image, second for image with delineation for explanation, and third for image with point annotation) to visualize the crowd task. (**c**) Displayed after the qualification phase to recapitulate cell features before solving the task. Here, 1 in yellow: astrocytes (red nucleus, blue cytoplasm, star-shaped), 2 in red: endothelial cells (red nucleus, pale blue cytoplasm, spindle-like), 3 in green: other non-neoplastic cells (red nucleus), and 4 in magenta: tumor cells (white nucleus, blue cytoplasm, star-shaped).

In the second iteration (PE2), we optimized comprehensibility of the crowd instructions and analyzed the influence of the crowd size on the quality of the aggregated solution. For the instructions, a questionnaire showed that (1) the motivation of about 50% of the school students increased by the explanation of the medical relevance, (2) tool usability was important for motivation, and (3) the task definition needed adjustments. For about 80% of the students, it was clear what the requested activity was, whereas the others struggled with the task, particularly with the aspect of similarity of cells. This systematic assessment resulted in minor adoptions of the example images, including, additional representation of the same image showing dots in addition to the outlines as we observed that some participants placed points around the cells to mimic their borders instead of clicking at the cell nuclei. A further observation identified in PE2 was that some contributors misclassified pre-existing cells with the same marker-constellation, yet clearly different morphology of cells, such as neurons or components of blood vessels. The obvious commonality with astrocytes (the red nuclear ATRX signal), but otherwise fundamentally different composition (cell shape, position, or size) required additional explanations. Therefore, we included a figure depicting important cell features to call crowdworker’s attention to difficulties of the astrocyte task (Fig. 2c), displayed directly after the qualification phase. As for the optimal crowd size, PE2 also showed that aggregating the annotations of more than 6–7 participants did not further increase the accuracy. This was in agreement with own published results from previous experiments with a crowd of medical students^16^. Based on these results and to reduce the risk of bias due to low performers by introducing a small “reserve”, we used a crowd size of at least ten contributors.

PE3 was necessary to determine an suitable number of MSIs given that task complexity required advanced crowd instructions on one side, while on the other hand published recommendations clearly suggested to consequently limit the time for task duration. In PE3, we observed an approximate processing time of 4 min per MSI (astrocyte annotation) respectively 8 min per MSI (tumor cell annotation). Based on this result, we included four MSIs for the astrocyte task (i.e. in total 16 larger tiles) and two MSIs for tumor task (i.e. in total 32 intermediate sized tiles) to keep within the aimed total processing time of 15–20 min. Further adjustment based on volunteers’ feedback improving the third representation (dot and outline) of the example images by focusing on the dot (final version of example images: Fig. 2b).

In contrast to PE1–3, the last experiment (PE4) was designed in AMT to validate the setting in a real-world scenario. Therefore, a direct contact to the workers or questionnaires for detailed feedback, were not possible anymore. Thus, our conclusions were based on observations in the obtained annotations. We addressed misclassifications by updating the crowd instruction (Fig. 2c), changed explanations towards plain English, and avoided logical operations. We here included a video (Supplementary Video S1) and icons (Fig. 2a) that allow an accessible label explanation. The sum of results from those consecutive pilot experiments led us to the final task design.

#### Feasibility

We applied the final task design to our main data set, which was considered for ML training. Therefore, we split the data set into 30 astrocyte tasks and 37 tumor cell tasks on AMT. The crowd quickly labeled potential tumor cells in 74 MSIs and potential astrocytes in 163 MSIs (in hours). In comparison, the expert needed months (12,642 tumor cells and 440 astrocytes in 59 MSIs) for the ground truth used for crowdsourcing or in ML test set. Total cost for AMT were $2207.49 (including charges and vat). This results in expenses for AMT-based annotation of about $10 per MSI, compared to about $120 for about 30 min per MSI annotated by board-certified pathologists. As the tasks were immediately unavailable due to demand and solution, latency control was not necessary in contrast to others^44,45^.

**Figure 3:**
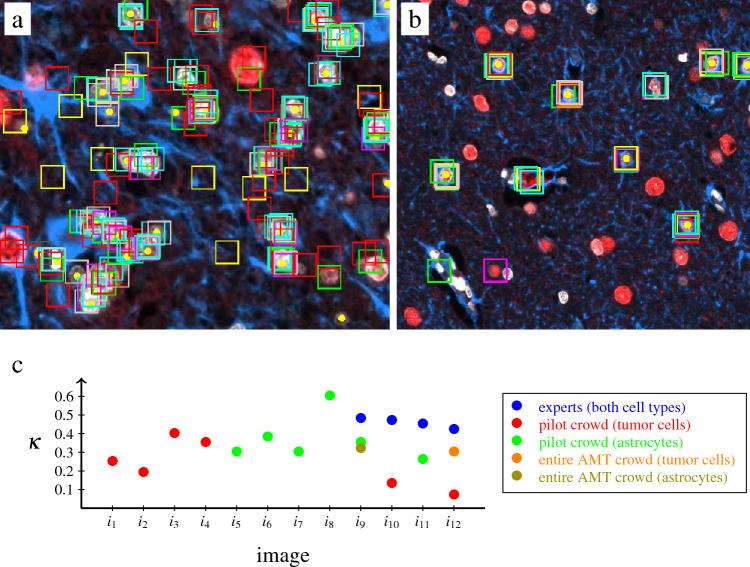
Performance of individual crowdworkers. (**a**) Tumor task; (**b**) astrocyte task. Yellow dots show ground truth, rectangles are bounding boxes around the crowd’s point annotations (different colors for each worker). (**c**) Fleiss $\kappa$ to assess inter-rater reliability. The entire Amazon Mechanical Turk (AMT) crowd annotated only a small tile of image $i_9$ and $i_{12}$ (ground truth-image), whereas the pilot crowds and experts labeled the entire multiplex immunofluorescence image.

Figure 3a,b show the annotations on the ground truth-image of 10 workers. The majority found the correct astrocytes and missed some tumor cells. Cells wrongly labeled as astrocytes e.g., neurons showed a similarity to astrocytes by sharing an ATRX positive nuclei. Other false positives concerned vessels, near which usually astrocytes stay to form the blood brain barrier, but also other cell types, where GFAP is present in the vicinity but does not belong to the cell. As the cells labeled by several workers were almost correct, this figure clearly shows the strongness of an answer aggregation. The inter-rater reliability was consistent with the visual evaluation. It was determined between non-experts on the ground truth-image of the AMT crowd with 270 workers for astrocytes (Fleiss $\kappa = 0.32$), 318 workers for tumor cells ($\kappa = 0.30$) and 10 further MSIs on pilot crowds, as well as between three experts for four MSIs and both cell types showing a $\kappa$ of 0.46 on average (Fig. 3c). Individual labels showed a median $F_1$ score of 0.47 for astrocytes and 0.73 for tumor cells considering the ground truth-tiles. As expected, a few workers clicked only on some cells probably to quickly finish the task. In contrast, we observed high-performers that provided annotations close to expert ones.

Aggregated crowd annotations compared to an expert (Table 2) showed a quite high PPV in general and reduced for WV in ground truth-tiles (note: the ground truth-image was used for WV-construction). MV resulted for astrocytes in a TPR of 0.48, a PPV of 0.90, and a $F_1$ score of 0.62 combining the entire ground truth, as well as for tumor cells in a TPR of 0.47, a PPV of 0.97, and a $F_1$ score of 0.63. This allows a first assumption that workers developed an understanding how the correct target structures look like.

**Table 2:** Crowd’s feasibility (weighted vote and majority vote of crowd annotations) on a ground truth-image and scattered tiles annotated by expert.

	Astrocytes							Tumor cells						
	*c*	Weighted vote			Majority vote			*c*	Weighted vote			Majority vote		
		TPR	PPV	$F_1$	TPR	PPV	$F_1$		TPR	PPV	$F_1$	TPR	PPV	$F_1$
Ground truth-image	7	0.26	1.0	0.44	0.46	0.97	0.56	38	0.46	1.0	0.63	0.45	0.98	0.60
Ground truth-tiles	23	0.52	0.50	0.51	0.57	0.62	0.59	96	0.75	0.78	0.77	0.63	0.82	0.71

TPR refers to the true positive rate, PPV to positive predictive value, *c* to the number of cells, and $F_1$ to the $F_1$ score.

### Machine learning

We trained YOLOv5 and YOLOv8-based CNNs in nine variants with varying image representation on aggregated crowdlabels and tested them on expert annotations. To reach a comparable training for each variant, we specified a minimum number of 1000 training epochs. The observed number of iterations for the training to converge was with 200–500 epochs on average below this threshold. The YOLOv8-based networks achieved $F_1$ scores of 0.61–0.69 averaged over both cell types, which was primarily due to low scores for astrocytes (Table 3) AP50 was in the range of 0.15–0.27 as a larger fraction of overlapping bounding boxes had an intersection of union between 0.35 and 0.50 (intersection over union distribution given in Supplementary Fig. 1. The scores of YOLOv5-based networks are given in Supplementary Table 2, together with a qualitative comparison with Faster RCNN on Var2 in Supplementary Fig. 2.

**Table 3:** Comparison of machine learning variants in YOLOv8.

	Astrocytes					Tumor cells					Mean		
	TPR	PPV	AP50	AP@	$F_1$ score	TPR	PPV	AP50	AP@	$F_1$ score	AP50	AP@	$F_1$ score
Var1	0.52	0.27	0.14	0.01	0.35	0.55	0.90	0.16	0.03	0.68	0.15	0.02	0.52
Var2	0.57	0.36	0.20	0.03	0.44	0.53	0.93	0.17	0.03	0.67	0.19	0.03	0.56
Var3	0.33	0.36	0.09	0.01	0.35	0.50	0.93	0.23	0.05	0.65	0.16	0.03	0.50
Var4	0.40	0.63	0.21	0.03	0.49	0.51	0.92	0.17	0.03	0.66	0.19	0.03	0.58
**Var5**	**0.59**	**0.44**	**0.25**	**0.04**	**0.51**	**0.54**	**0.76**	**0.30**	**0.06**	**0.63**	**0.27**	**0.05**	**0.57**
Var6	0.55	0.51	0.23	0.04	0.53	0.50	0.78	0.25	0.04	0.61	0.24	0.04	0.57
Var7	0.56	0.38	0.19	0.03	0.45	0.57	0.86	0.22	0.04	0.69	0.20	0.03	0.57
Var8	0.42	0.37	0.13	0.02	0.40	0.51	0.86	0.24	0.04	0.64	0.19	0.03	0.52
Var9	0.52	0.33	0.14	0.02	0.40	0.51	0.88	0.31	0.07	0.65	0.22	0.04	0.53

TPR refers to the true positive rate and PPV to positive predictive value. TPR, PPV, and $F_1$ score are based on an intersection over union of at least 0.35 between overlapping bounding boxes. AP50 and AP@ referring to AP@$[0.5:0.05:0.95]$ include the average precision. Var1–4 contain the markers DAPI, ATRX, and GFAP; Var5–6 DAPI and GFAP; Var7–8 DAPI, GFAP and other; Var9 DAPI, ATRX, GFAP, and other; also differing in their colors and intensities (details in Fig. 1).

Best variant highlighted in bold.

**Figure 4:**
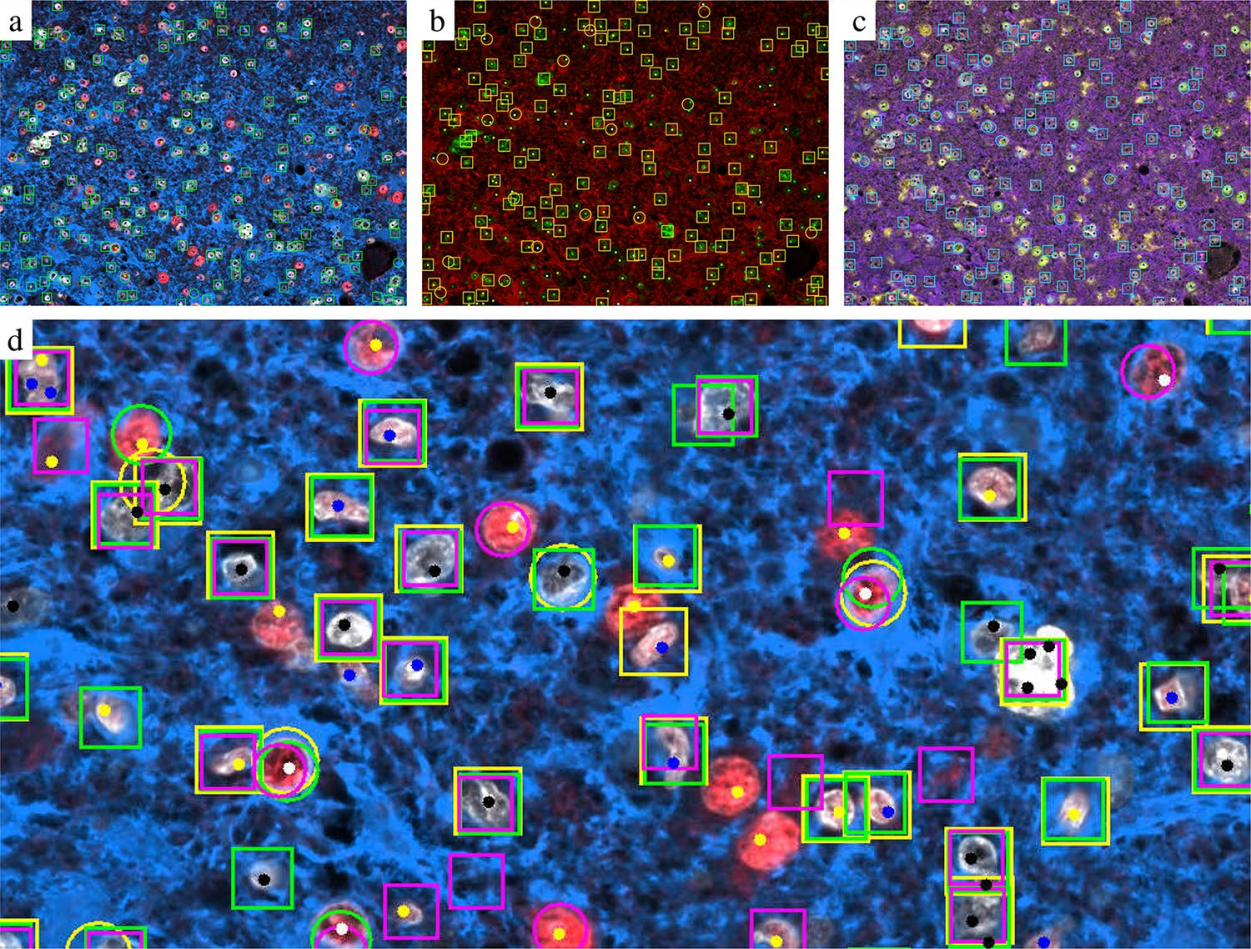
Detections of YOLOv5 convolutional neuronal networks for astrocytes (circles around pixels predicted as cell centers) and tumor cells (rectangles). (**a**) Var1; ground truth represented by yellow (astrocyte), black (tumor), blue (other cell) dots. (**b**) Var5; ground truth represented by white (astrocyte), green (tumor), yellow (other cell) dots. (**c**) Var9; ground truth represented by red (astrocyte), black (tumor), and blue (other cell) dots. (**d**) Var1 (green), Var5 (yellow), Var9 (magenta). Ground truth represented by black (tumor), white (astrocytes), blue (immune cells), and yellow (others) dots.

The PPV showed that most of the detected tumor cells were true positive. This can be shifted by adopting the confidence threshold (data not shown). Var1 used the same markers and colors as the crowd, Var2 differed in the color intensity, but the CNN of Var2 showed a slightly better quality. Var7–9 including additional markers received no clear improvement of the quality. A comparison between Var1, 5, and 9 on individual cells is illustrated in Fig. 4. It shows that especially Var9 detected other cells like neurons as astrocytes and areas without nuclei as tumor cells. In Var9, the number of false positive astrocytes was increased. We assume that mixed colors due to the overlap between the channel could complicate assignment of markers. Several false positive tumor cells detected by Var1 and Var5 had a reduced ATRX signal in their nuclei. Var5 surprisingly achieved the best results especially clearly better than Var1. Since Var5 used a color scheme without the important ATRX information, this shows the importance of the cell morphology. Our results showed that a CNN trained on aggregated crowdlabels could detect tumor cells and astrocytes and reduced marker information led to success.

## Discussion

This study focused on task design as prerequisite for reliable application of crowdsourced data in ML in the context of labeling astrocytes and tumor cells in high-grade IDH1-mutated astrocytoma. We showed feasibility of the approach and discuss the implications of the results for tumor microenvironment analysis beyond IDH1 mutated glioma, specifically for potential applications in IDH wildtype glioblastoma.

Starting from general recommendations^5^, we developed an adaptable task design focusing on tile size, number of MSIs, crowd size, and especially on crowd instruction. Optimization of tile size is an important step. Workers and pathologists may consider information on the microenvironment when facing larger tiles, e.g., “calibrating” their assessment based on neighboring tissue structures. In contrast, smaller tiles lack this advantage, but reduce the common risk to lose focus on the important structures. In our experience, most pathologists prefer to work on annotations in the context of metadata (such as age, localization, medical history) and larger tiles, ideally with a zoom function to change magnification. Our results indicate that non-experts tend to concentrate on specific cells without significant consideration of adjacent structures, reflected by better performance on smaller tiles. Further, our observations confirm the importance of graphical instructions, and explanations in simple language^5^. Interestingly, the motivation of school students with an interest in the field of computational biology could be increased by providing medical background information. Due to the setting of crowdsourcing on AMT, without an option to obtain feedback from crowdworkers, we could not study the effect of motivation in the final experiment.

As expected considering the task difficulty, we found a relatively low agreement between crowdworkers, but $\kappa$-values were only slightly lower than between three experts. In the field of pathology, inter-rater inconsistency is a general effect that needs to be addressed in model generation, e.g., Bertram et al. reported a considerable expert-related disagreement for mitosis labeling^46^. Tabata et al. described Cohens $\kappa$ values between 0.6 and 0.9 for mitosis labeling between different experts and intra-rater values based on different visualization tools between 0.59 and 0.94^47^. Ji et al. described a moderate concordance between the five experts (Fleiss $\kappa = 0.6$) in the context of bounding boxes^48^.

Our final task design resulted in aggregated $F_1$ scores that indicated feasibility for crowdsourcing in this setting. In our specific use case, the complexity was mainly due to exceptional morphological similarity between the classes of cells for annotation. An additional level of complexity was caused by almost inevitable technical limitations, such as remaining “diffuse” staining background, even after thorough technical optimization of staining or imperfect color deconvolution due to overlapping fluorophore emission spectra. In these conditions, the signal can not unambigiously assigned to a given marker, unless an evaluator has specific domain knowledge. Hence, we reduced the number of markers for crowdsourcing to the bare minimum. Observed errors, like incorrect labeling of cells with similar marker expression as astrocytes, but (for experts) obviously not part of this class, were in line with the notion that lack of domain knowledge in non-expert crowds can reduce accuracy^49^. In contrast to resource-intensive approaches such as an iterative pipeline involving experts in annotation revision^26^, we propose a scalable concept with answer aggregation—the gold standard for quality control. Whereas availability of trained pathologists is limited, crowdlabels are scalable once the system works. To deal with outliers and weaknesses, answer aggregation and noise handling can work well to mitigate problems introduced by single workers^16,18,50^. Surprisingly, in our setting, MV performed better than WV. As we could not study whether the performance of a single worker strongly varies between images inside a task, we rely on literature data that showed a stable performance of individuals in a single histology task^16^. Therefore, we assume a better behavior of WV by including more cells to measure the weights, distributing these cells over the entire task, or adopting the definition of the WV.

Overall, we showed feasibility for a scalable, robust, and standardized cell detection which requires computational methods. We generated training data with our crowdsourcing approach, evaluated the crowd’s performance based on a ground truth-image and ground truth-tiles, and then additionally tested the crowdsourced labels in ML-tools using an independent, expert-annotated test set. Often, crowdlabels are used for training^1,3^ or as data augmentation. This clearly outperforms models purely trained on expert annotations^2^. We trained nine YOLOv5 and YOLOv8-CNNs exclusively with crowdsourcing labels in order to reserve the precious and accurate expert annotations for testing. The performance of these networks confirmed that the quality of crowdsourcing was sufficient to provide adequate training data in this case based on TPR, PPV, and $F_1$ score using a slightly relaxed definition of the required intersection over union of at least 0.35 for overlapping bounding boxes. Classical measurements like average precision showed the challenges of bounding boxes related to a task focused on largely variable shapes and the position of cell nuclei in relation to the visible cell processes. Besides the detection of clearly distinguishable individual cells, we also observed that a model may predict overlapping bounding boxes, classified as astrocyte and tumor cell, which seem to refer to the same cell. A possible scenario is the biological existence of only one cell which is erroneously predicted as two cells of different type, which may be driven by morphological variations such as pleomorphism or surrounding staining. A second scenario corresponds biologically to the unique mechanism of diffuse glioma invasion with mutually interacting pre-existing non-neoplastic and malignant cell with astrocytic features, and can actually reflect physically overlapping cells of different cell types with eventually interlacing cell processes. This issue was related to machine learning and not to crowdsourcing as we intentionally simplified the task design, for example to consider point annotations for the crowd, and to use two separate tasks for astrocytes and tumor cells.

In context of most downstream analyses, we consider the approach feasible, as predicted cells are mainly used as input of subsequent analyses like neighborhood metrics, and therefore cell centers but not bounding boxes themselves are further processed. We assume that an adjusted WV (see above) and oversampling could further improve our predictions, especially increase the scores for astrocytes. Besides, the effect of our two-tier approach that a model included a single cell type and secondly both cell types can be considered. For crowdsourcing, it is not reasonable to combine both cell types in a single task as the increased complexity usually decreases the quality^15,17^. However, two independent tasks (by dealing with low astrocyte frequencies in tumor core) can include the same images such that the final model can be trained directly on both cell types. Here, we did not focus on maximizing the detection quality of the CNN, instead our goal was to test whether the crowdlabels enable reasonable training of a CNN. In a further study, it could be helpful towards a high-accurate CNN to adopt the CNN architecture and analyze its effect. We tested the effect of different color schemes showing that variants with reduced marker information (Var5–6) performed better. This has the benefit that the method is applicable to similar multiplex stainings sharing common markers like ATRX and GFAP, but the choice of additional markers (e.g., other immune cells than macrophages) should not influence the cell prediction.

This important finding also indicated a potential for a transfer learning approach. For this, labels were prepared based on tumor-specific markers (e.g., ATRX-loss in high-grade IDH1 astrocytoma as represented in Var1). Then, a CNN was trained with these labels and successfully applied on images lacking these tumor-specific information (Var5). This opens the opportunity to expand the use of this CNN to the much more common glioblastoma (IDH-wildtype) without those markers. Astrocytoma images can be annotated in Var1, the model can be trained with these labels in Var5, and then applied to glioblastoma in the same color scheme. This is relevant for further research in the field, given that even experts can occasionally hardly distinguish between massively reactive astrocytes and tumor cells^20^. Our results clearly indicate a potential for successful transfer of our method towards IDH1 wildtype glioma; however, validation is limited due to the current lack of specific histopathological tumor-cell markers that can be combined with other markers in multiplexed immunhohistochemistry. The very rare supratentorial high grade gliomas with H3G34 mutations and the slightly more frequent, however, also rare, diffuse midline glioma (DMG) with H3K27M mutation would in principle be potential application fields. However, the rare occurrence (H3G34 mutations) and specific morphology (H3K27M) limit feasibility and medical need for this application.

In summary, we developed a task design which was feasible for complex cell annotations of reactive astrocytes and tumor cells at single-cell level. Our results indicated that CNNs trained with aggregated crowdlabels are suitable for glioma microenvironment analysis. Together with first evidence for successful application of transfer learning in tumors lacking specific immunohistochemical markers, this indicates considerable potential for this emerging scientific field and supports the need for further development of crowdsourcing and advanced image analysis in brain tumor research.

### Supplementary Information

Supplementary Figure 1.

Supplementary Figure 2.

Supplementary Table 1.

Supplementary Table 2.

Supplementary Video S1.

## Data Availability

The task design is available in the manuscript, the video in Supplementary Material S1. Images will be made publicly available through an appropriate platform like https://www.openmicroscopy.org/ to be downloaded for academic purpose.
