# Supplementary material for: Task design for crowdsourced glioma cell annotation in microscopy images
Source: Sci Rep. 2024 Jan 23;14:1965. doi: 10.1038/s41598-024-51995-8 (PMC10805723; doi:10.1038/s41598-024-51995-8)
Supplement: Supplementary file 1 — Supplementary Figure 1. [file 41598_2024_51995_MOESM1_ESM.pdf]

Supplementary Figure 1 – Task design for crowdsourced glioma cell annotation in microscopy images

Svea Schwarze[1,+], Nadine S. Schaadt[1,+], Viktor M. G. Sobotta[2], Nicolai Spicher[2], Thomas Skripuletz[3], Majid Esmaeilzadeh[4], Joachim K. Krauss[4], Christian Hartmann[1], Thomas M. Deserno[2], and Friedrich Feuerhake[1,5,*]

[1]Department of Neuropathology, Institute for Pathology, Hannover Medical School, Hannover, Germany
[2]Peter L. Reichertz Institute for Medical Informatics of TU Braunschweig and Hannover Medical School, Braunschweig, Germany
[3]Department of Neurology, Hannover Medical School, Hannover, Germany
[4]Department of Neurosurgery, Hannover Medical School, Hannover, Germany
[5]Department of Neuropathology, University Clinic Freiburg, Hannover, Germany
[*]Feuerhake.Friedrich@mh-hannover.de
[+]these authors contributed equally to this work

ABSTRACT

Results – Machine learning

Supplementary Figure 1 shows the distribution of overlapping are between predicted and ground truth bounding boxes.

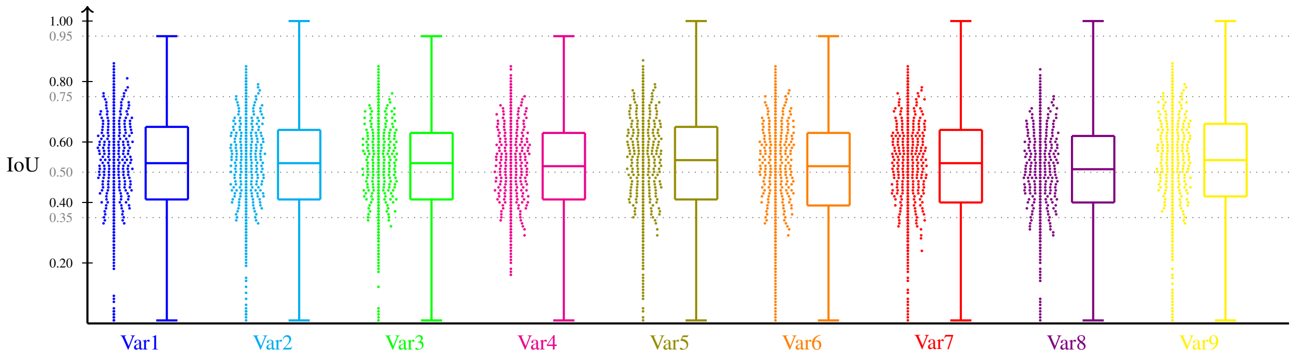

Figure 1. Distribution of intersection over union (IoU) between YOLOv8 bounding boxes and their overlapping ground truth bounding boxes for all variants. The box plot shows the minimum, maximum, median, lower and upper quartile. Each dot represents 20 overlapping bounding boxes.
