# Supplementary material for: Task design for crowdsourced glioma cell annotation in microscopy images
Source: Sci Rep. 2024 Jan 23;14:1965. doi: 10.1038/s41598-024-51995-8 (PMC10805723; doi:10.1038/s41598-024-51995-8)
Supplement: Supplementary file 2 — Supplementary Figure 2. [file 41598_2024_51995_MOESM2_ESM.pdf]

Supplementary Figure 2 – Task design for crowdsourced glioma cell annotation in microscopy images

Svea Schwarze[1,+], Nadine S. Schaadt[1,+], Viktor M. G. Sobotta[2], Nicolai Spicher[2], Thomas Skripuletz[3], Majid Esmaeilzadeh[4], Joachim K. Krauss[4], Christian Hartmann[1], Thomas M. Deserno[2], and Friedrich Feuerhake[1,5,*]

[1]Department of Neuropathology, Institute for Pathology, Hannover Medical School, Hannover, Germany
[2]Peter L. Reichertz Institute for Medical Informatics of TU Braunschweig and Hannover Medical School, Braunschweig, Germany
[3]Department of Neurology, Hannover Medical School, Hannover, Germany
[4]Department of Neurosurgery, Hannover Medical School, Hannover, Germany
[5]Department of Neuropathology, University Clinic Freiburg, Hannover, Germany
[*]Feuerhake.Friedrich@mh-hannover.de
[+]these authors contributed equally to this work

ABSTRACT

Results – Machine learning

Supplementary Figure 2 qualitatively compares the predictions between Faster RCNN and YOLOv8.

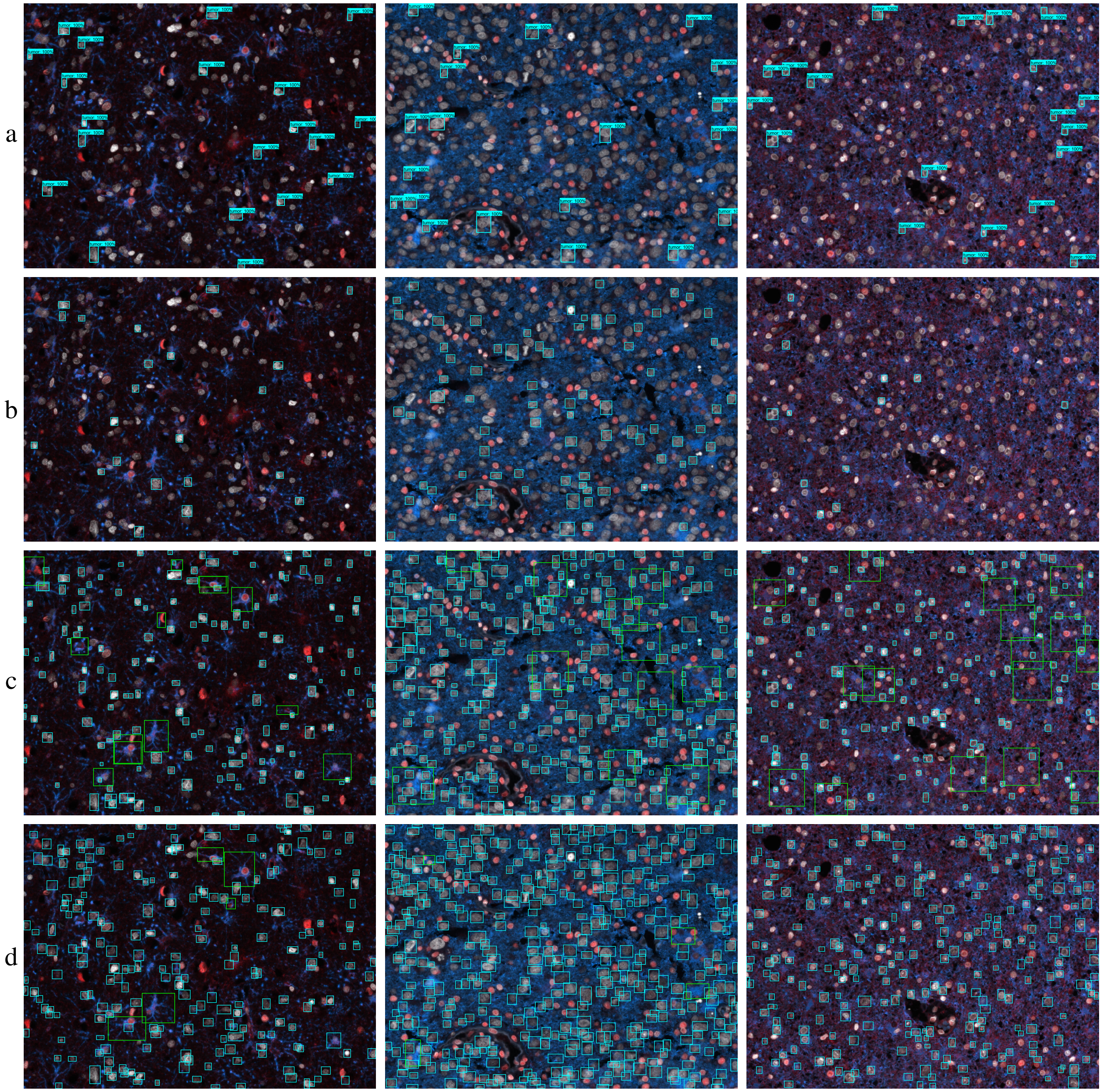

Figure 2. Comparison between Faster RCNN and YOLOv8 predictions (Var2). Bounding boxes in cyan refer to tumor cells, in green to astrocytes. a: Faster RCNN, b: YOLOv8 with a confidence threshold of 0.90, c: YOLOv8 with a confidence threshold of 0.25, d: ground truth.
