# Supplementary material for: Task design for crowdsourced glioma cell annotation in microscopy images
Source: Sci Rep. 2024 Jan 23;14:1965. doi: 10.1038/s41598-024-51995-8 (PMC10805723; doi:10.1038/s41598-024-51995-8)
Supplement: Supplementary file 3 — Supplementary Table 1. [file 41598_2024_51995_MOESM3_ESM.pdf]

Supplementary Table 1 – Task design for crowdsourced glioma cell annotation in microscopy images

Svea Schwarze[1,+], Nadine S. Schaadt[1,+], Viktor M. G. Sobotta[2], Nicolai Spicher[2], Thomas Skripuletz[3], Majid Esmaeilzadeh[4], Joachim K. Krauss[4], Christian Hartmann[1], Thomas M. Deserno[2], and Friedrich Feuerhake[1,5,*]

[1]Department of Neuropathology, Institute for Pathology, Hannover Medical School, Hannover, Germany
[2]Peter L. Reichertz Institute for Medical Informatics of TU Braunschweig and Hannover Medical School, Braunschweig, Germany
[3]Department of Neurology, Hannover Medical School, Hannover, Germany
[4]Department of Neurosurgery, Hannover Medical School, Hannover, Germany
[5]Department of Neuropathology, University Clinic Freiburg, Hannover, Germany
[*]Feuerhake.Friedrich@mh-hannover.de
[+]these authors contributed equally to this work

ABSTRACT

Methods – Machine learning

Supplementary Table 1 shows an estimation about computational complexity of the single processing steps (for machine learning processes, we used the required time for training instead of complexity).

Table 1. Computational complexity of each image analysis step and time to process machine learning tools. The process steps are described in Methods – Machine learning section of main document. Training of the models was performed for 1000 epochs.

Process step	Complexity	Time
Background removal	O(n)	–
Flood-fill algorithm	O(n)	–
Preliminary models (single cell type)	–	∼1.5 hours
Combining both results	O(n)	–
Final model	–	∼4 hours
