# Supplementary material for: Task design for crowdsourced glioma cell annotation in microscopy images
Source: Sci Rep. 2024 Jan 23;14:1965. doi: 10.1038/s41598-024-51995-8 (PMC10805723; doi:10.1038/s41598-024-51995-8)
Supplement: Supplementary file 4 — Supplementary Table 2. [file 41598_2024_51995_MOESM4_ESM.pdf]

Supplementary Table 2 – Task design for crowdsourced glioma cell annotation in microscopy images

Svea Schwarze[1,+], Nadine S. Schaadt[1,+], Viktor M. G. Sobotta[2], Nicolai Spicher[2], Thomas Skripuletz[3], Majid Esmaeilzadeh[4], Joachim K. Krauss[4], Christian Hartmann[1], Thomas M. Deserno[2], and Friedrich Feuerhake[1,5,*]

[1]Department of Neuropathology, Institute for Pathology, Hannover Medical School, Hannover, Germany
[2]Peter L. Reichertz Institute for Medical Informatics of TU Braunschweig and Hannover Medical School, Braunschweig, Germany
[3]Department of Neurology, Hannover Medical School, Hannover, Germany
[4]Department of Neurosurgery, Hannover Medical School, Hannover, Germany
[5]Department of Neuropathology, University Clinic Freiburg, Hannover, Germany
[*]Feuerhake.Friedrich@mh-hannover.de
[+]these authors contributed equally to this work

ABSTRACT

Results – Machine learning

Supplementary Table 2 shows the scores of all variants for YOLOv5.

	Astrocytes					Tumor cells					Mean		
	TPR	PPV	AP50	AP@	F_1 score	TPR	PPV	AP50	AP@	F_1 score	AP50	AP@	F_1 score
Var1	0.58	0.39	0.22	0.03	0.47	0.57	0.89	0.20	0.04	0.70	0.21	0.03	0.59
Var2	0.54	0.44	0.25	0.04	0.48	0.54	0.92	0.17	0.03	0.68	0.21	0.04	0.58
Var3	0.48	0.48	0.20	0.03	0.48	0.54	0.93	0.17	0.04	0.68	0.19	0.04	0.58
Var4	0.48	0.33	0.14	0.02	0.39	0.54	0.93	0.19	0.04	0.68	0.16	0.03	0.54
Var5	0.58	0.34	0.13	0.02	0.42	0.46	0.80	0.33	0.06	0.58	0.23	0.04	0.50
Var6	0.41	0.53	0.17	0.03	0.47	0.51	0.77	0.29	0.05	0.62	0.23	0.04	0.55
Var7	0.48	0.44	0.20	0.03	0.46	0.50	0.91	0.32	0.07	0.64	0.26	0.05	0.55
Var8	0.59	0.30	0.23	0.03	0.40	0.54	0.87	0.31	0.06	0.67	0.27	0.05	0.54
Var9	0.39	0.56	0.18	0.02	0.46	0.51	0.88	0.29	0.06	0.65	0.24	0.04	0.56

Table 2. Comparison of machine learning variants in YOLOv5. TPR refers to the true positive rate and PPV to positive predictive value. TPR, PPV, and F_1 score are based on an intersection over union of at least 0.35 between overlapping bounding boxes. AP50 and AP@ referring to AP@[0.5 : 0.05 : 0.95] include the average precision. Differences between Var1–9 in contained marker, colors, and intensities are given in details in Figure 1 of main text.
